# Healthcare professionals' experiences of required competencies in mentoring of interprofessional students in clinical practice: A systematic review of qualitative studies

**DOI:** 10.1111/jan.16347

**Published:** 2024-08-04

**Authors:** Jonna Juntunen, Anna‐Maria Tuomikoski, Sari Pramila‐Savukoski, Veera Kaarlela, Anna‐Leena Keinänen, Maria Kääriäinen, Kristina Mikkonen

**Affiliations:** ^1^ Research Unit of Health Science and Technology, Faculty of Medicine University of Oulu Oulu Finland; ^2^ Wellbeing Services County of North Ostrobothnia Oulu University Hospital Oulu Finland; ^3^ University of Applied Science Oulu Finland; ^4^ Medical Research Center Oulu Wellbeing Services County of North Ostrobothnia Oulu Finland

**Keywords:** clinical placements, clinical supervision, mentors, multi‐professional education, systematic review

## Abstract

**Aim:**

To synthesize evidence on healthcare professionals' experiences of competencies in mentoring undergraduate healthcare, social care and medical students during their interprofessional clinical practice.

**Design:**

This review was conducted by the JBI methodology for systematic reviews of qualitative evidence.

**Methods:**

Studies were included if they were based on the phenomenon of interest and used qualitative or mixed methods (qualitative share). The included studies were critically appraised using the standardized JBI Critical Appraisal Checklist. Qualitative research findings were extracted and synthesized using the meta‐aggregation approach.

**Data Sources:**

Five databases (CINAHL, PubMed, Scopus, Medic and ProQuest) were systematically searched from each database's inception on 28 June 2023.

**Results:**

A total of 5164 studies were initially screened, and 25 were identified for inclusion in this review. Three synthesized findings were identified: competencies related to (1) preparing for and developing interprofessional clinical practice, (2) supporting the learning process in interprofessional clinical practice and (3) creating an interprofessional mentor identity.

**Conclusion:**

Although competent mentors are essential to implementing and developing interprofessional clinical practice, some mentors find interprofessional mentoring challenging. High‐quality interprofessional mentoring requires specific competence that differs from profession‐specific and individual mentoring.

**Implications for the Profession and/or Patient Care:**

To ensure that interprofessional clinical practice is of high quality and strengthens students' professional and interprofessional growth, special attention should be given to mentors' interprofessional mentoring competence, and a range of opportunities and organizational structures should be provided for competence development.

**Impact:**

This systematic review provides insights into the specific competencies required for interprofessional mentoring. These findings can support healthcare professionals, educators and policymakers in developing interprofessional clinical practice and mentoring competence.

**Reporting Method:**

This review adhered to the Preferred Reporting Items for Systematic Reviews and Meta‐Analysis (PRISMA) statement and ENTREQ reporting guidelines.

No patient or public contribution.

**What does this paper contribute to the wider global clinical community?**

- The need for interprofessional competence of future healthcare professionals is undisputed.
- Interprofessional clinical practice contributes to the professional growth of students from different professions and strengthens their interprofessional competencies.
- The results of this systematic review will guide best practices and guidelines for developing mentors' interprofessional mentoring competence, interprofessional clinical practice and mentor education.

## 1 INTRODUCTION

In the last decade, social and healthcare environments have become more complex and multifunctional (Spaulding et al., 2021). Patients' life situations require increasingly more holistic attention and extensive competencies from healthcare professionals (World Health Organization, 2019), which is not situated in a single professional field (Khalili et al., 2019). Interprofessional collaboration is crucial for facilitating systems integration and improving healthcare outcomes (Spaulding et al., 2021), quality of patient care and experiences, staff work satisfaction and engagement (Pomare et al., 2020). Interprofessional collaboration is defined as a process in which healthcare professionals from different professions work together to provide people‐centred care (Khalili et al., 2019). World Health Organization (2010) has stressed the importance of interprofessional education (IPE) for developing a collaborative practice‐ready health workforce (World Health Organization, 2010). IPE concludes in practice as a communication and decision‐making process that focuses on common goals, identifies and respects individual strengths and differences, is people centred and aims to improve health outcomes (Barr et al., 2017).

## 2 BACKGROUND

IPE is a teaching model or an education approach where ‘learners from two or more professions learn about, with and from each other, to improve collaboration and the quality of care and services’ (World Health Organization, 2010). Previous reviews have demonstrated the effectiveness of IPE in improving learners' knowledge, skills (Hopkins et al., 2022; Marion‐Martins & Pinho, 2020) and behaviour of interprofessional collaborative competencies (Hopkins et al., 2022; Marion‐Martins & Pinho, 2020; Mattiazzi et al., 2024) as well as their understanding of their role and scope in practice (Hopkins et al., 2022). IPE is grounded in educational theories, for example, adult learning, community of practice, constructivist, learning by doing and learning‐is‐doing theories and particularly recently, the socio‐cultural theory of learning, transformative learning and contact‐learning theories (Mattiazzi et al., 2024). For undergraduate healthcare, social care and medical students (afterwards students), IPE has recently been implemented in various learning environments, like synchronously engaged with face‐to‐face and online learning (Bridges et al., 2020), simulation (Marion‐Martins & Pinho, 2020), online (Powers & Kulkarni, 2021) and clinical practice (Kent et al., 2017; Mattiazzi et al., 2024) settings.

Students' clinical learning relies intensely on clinical practice in various healthcare settings and specifically on mentoring from individual healthcare professionals, who have traditionally focused on mentoring students from their profession (Nursing and Midwifery Council, 2023; O'Brien et al., 2019; Tuomikoski et al., 2018). Mentoring within different professions varies widely globally, and only a few countries, such as the United Kingdom, Ireland and the United States, provide mandatory training for mentors (Mikkonen et al., 2022). For example, in 2008, the Nursing and Midwifery Council defined the requirements for acting as a mentor or sign‐off mentor (Nursing and Midwifery Council, 2023). These terms were replaced by practice supervisor, practice and academic assessor in 2018. Furthermore, other terms such as facilitator, supervisor and preceptor are used interchangeably by mentors responsible for mentoring students in clinical practice (Tuomikoski et al., 2020). In traditional clinical practice, mentors are responsible for pedagogical activities such as providing guidance, support and feedback to students in their learning process and assessing learning outcomes, together with providers from approved educational institutions (Nursing and Midwifery Council, 2023; Tuomikoski et al., 2020). The role of the mentor is described as a teacher, facilitator and role model (Mikkonen et al., 2020). Mentors support to enable students to develop a professional identity (Berndtsson et al., 2020) and to learn and safely achieve competence and autonomy in their professional role (Nursing and Midwifery Council, 2023). In order to fulfil the role of a mentor in clinical practice, it is necessary to have multiple mentoring competencies. From the perspective of nurse mentors, in previous research, mentors' competencies were explained by areas of (1) creating an interactive relationship with the student, (2) developing the mentor's characteristics and cooperation with stakeholders, (3) providing goal‐oriented mentoring, (4) supporting students' development to nurse profession and (5) supporting the student's learning process (Mikkonen et al., 2020; Tuomikoski et al., 2018).

The problem of this traditional clinical practice has long been seen in the professional silos, in which education mainly focuses on profession‐specific competencies, and students are practising separately from each other (Mink et al., 2021). However, IPE in clinical practice settings is recognized as an ideal for students to learn the competencies required to effectively work with professionals from different disciplines (Hopkins et al., 2022). As a result, interprofessional clinical practices for interprofessional student teams are increasing (Kent et al., 2017). Interprofessional clinical practice may involve different combinations of medical, nursing, pharmacy or allied health profession undergraduate students, and their practice level could vary from novice to advanced practitioner (Kent et al., 2017). These interprofessional student teams under mentoring synchronize their clinical practice to deliver high‐quality, people‐centred, team‐based care (Shrader & Zaudke, 2018). As in a traditional clinical practice (Berndtsson et al., 2020; Tuomikoski et al., 2020), mentors are also acknowledged as essential to successful interprofessional clinical practice (Shrader & Zaudke, 2018).

The role of the mentor in interprofessional clinical practice is to facilitate the development of professional identity and profession‐specific competencies (Oosterom et al., 2019) and to provide interprofessional learning opportunities for students (Shrader & Zaudke, 2018). Typically, the students are mentored by their profession‐specific mentor and mentors from other professions, who facilitate the interprofessional student team process (Brewer & Flavell, 2020; Oosterom et al., 2019). Based on the different roles of traditional and interprofessional mentors, mentoring in interprofessional clinical practice requires specific mentoring competencies for mentors. Mentors must facilitate interprofessional learning and co‐teaching, focusing on the interprofessional competencies of values and ethics of collaborative practice, role understanding and interprofessional communication (Brewer & Flavell, 2020; Lie et al., 2016). Mentors need to be attuned to the dynamics of interprofessional learning, optimize learning opportunities and value each profession's distinctive experiences and expertise (Kent et al., 2017). As mentors have traditionally focused on mentoring students from their profession (O'Brien et al., 2019), they often find their role challenging as they need to adapt their mentoring strategies to interact with and guide the learning of students from different professions equally (Kent et al., 2017). In interprofessional clinical practice, mentors have also felt respectively unprepared to model collaborative practice and facilitate complex groups (Lie et al., 2016). Committed and trained mentors are critical to effective interprofessional clinical practice (Kent et al., 2017; Lie et al., 2016; Lim & Noble‐Jones, 2018).

The number of reviews related to IPE has increased over the last decade. For example, recent reviews have examined the effectiveness of IPE programmes (Reeves et al., 2016) in clinical practice (Mattiazzi et al., 2024) and simulation‐based educational settings (Marion‐Martins & Pinho, 2020), identified the contexts, mechanisms and outcomes of pre‐registration students' formal interprofessional clinical learning (Kent et al., 2017) and identified assessment tools in pre‐registration IPE (Almoghiraf et al., 2021). While increasingly mentors are mentoring interprofessional student teams in clinical practice (Kreider et al., 2022), and the significant role of mentors in interprofessional clinical practice for student learning outcomes is recognized (Kent et al., 2017; Lim & Noble‐Jones, 2018), there is a paucity of evidence concerning the competencies required of healthcare professionals in mentoring interprofessional students in clinical practice. Qualitative studies exist, but currently, there were no identified attempts to synthesize mentors' experiences of mentoring students during their interprofessional clinical practice.

## 3 AIM

This systematic review aimed to determine the best available evidence on healthcare professionals' experiences of competencies in mentoring undergraduate healthcare, social care and medical students during their interprofessional clinical practice. Healthcare professionals' experience of competencies comprehend the qualities, attitudes, values, knowledge, skills and performances (Le Deist & Winterton, 2005) necessary for mentoring students during their interprofessional clinical practice. The results of this systematic review will guide towards best practices and guidelines to be used in the development of interprofessional clinical practice and the education of mentors.

## 4 METHODS

This review was conducted by the JBI methodology for systematic reviews of qualitative evidence (Lockwood et al., 2020).

### 4.1 Search strategy

A three‐step search strategy was utilized in this review. The first step was an initial limited search of the database CINAHL with full text (EBSCO). The initial limited search aimed to analyse the text words in the titles and abstracts and the index terms used to describe the articles. Findings from the first step were used to identify keywords and index terms used in databases. A comprehensive search strategy was developed with the assistance of an information specialist based on identified keywords and index terms from first‐step findings.

In the second step, the search was undertaken across all included databases: CINAHL with full text (EBSCO), PubMed, Scopus, Medic and ProQuest on 28 June 2023. The search terms used in all databases are shown in Appendix 1. In the third step, the reference lists of all included studies were searched for additional studies. Following the search, all identified citations were imported into Covidence, where, after removing duplicates, a comprehensive database search of the literature yielded 5164 potential titles. Each title and abstract, and then the remaining full text, were screened by two independent reviewers, JJ and either A‐MT, SP‐S, VK, A‐LK or KM, for assessment against the review's inclusion criteria. Any disagreements of the reviewer were resolved by discussion or by a third reviewer. After reviewing the titles and abstracts, 5021 studies were excluded. The remaining 143 and 2 additional studies found through a reference search were assessed for eligibility. Of these, 120 were excluded based on the inclusion criteria. A total of 25 studies were finally included in this review. The entire search strategy for all databases is shown in Table 1.

**TABLE 1:** Flow chart of study selection process (Lockwood et al., 2020).

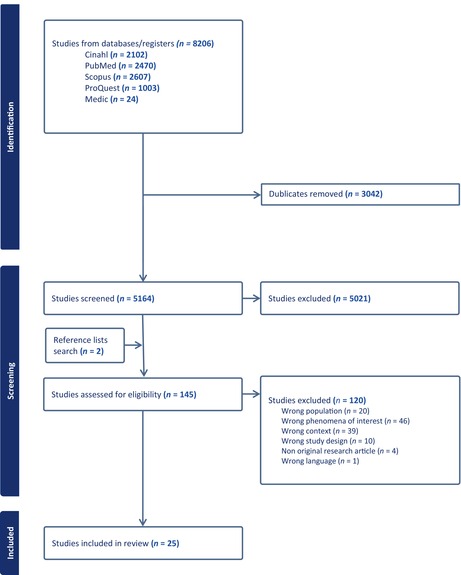

### 4.2 Study inclusion

Inclusion criteria were established based on the qualitative PICo format (Lockwood et al., 2020), where the population (P) were mentors of social care, healthcare or medical students in an interprofessional clinical practice; the phenomena of interest (I) were mentors' experiences related to competence in mentoring social care, healthcare or medical students during the interprofessional clinical practice and the context (Co) was a healthcare organization where social care, healthcare or medical students could complete an interprofessional clinical practice.

There were no date restrictions to get a broader perspective of the phenomenon of interest. Due to the research team's language skills, only studies written in English, Finnish or Swedish were considered for inclusion. Based on the phenomenon of interest, only qualitative or mixed methods (qualitative share) studies were included.

### 4.3 Assessment of methodological quality and data extraction

The remaining studies were critically appraised for methodological quality by two independent reviewers, JJ and either A‐MT, SP‐S, VK, A‐LK or KM, using the standardized JBI Critical Appraisal Checklist for Qualitative Research (Lockwood et al., 2020). Disagreements between the reviewers were resolved by discussion or by a third reviewer. The selected studies included in this review demonstrated good methodological quality (Appendix 2). All studies, regardless of methodological quality, underwent data extraction and synthesis. Qualitative data were extracted using the JBI‐QARI extraction tool. For each included study, the first author, year, country of origin, methodology, phenomena of interest, setting, participants and main findings were extracted (Table 2).

**TABLE 2:** Extracted data from studies included for the systematic review.

Study	Methodology design, methods, data analysis	Participants (population)	Setting	Phenomena of interest	Main findings included only qualitative findings of mentors. *Not‐supported findings (no illustration) are marked in italics*
Country					
Andermo et al. (2023) Sweden	A qualitative descriptive design	8 mentors (2 occupational therapists, 2 physiotherapists, 1 physician, 3 nurses) 35 students (18 nursing, 1 occupational therapist, 2 medical secretaries, 4 medicine, 10 social care) 8 mentors (nurses)	The collaborator Assessment Rubric (ICAR) tool assessed students' interprofessional competencies in different educational clinical contexts, including orthopaedic clinical interprofessional training wards, geriatric wards and academic primary healthcare centres. In these units, students completed their interprofessional practice and were mentored and assessed using ICAR by mentors	To explore the applicability of the Interprofessional Collaborator Assessment Rubric (ICAR) in different clinical education contexts from both student and mentor perspectives	3 of 4 sub‐themes supported by illustration were identified: (1) a relevant working tool for assessment; (2) a relevant assessment tool stimulated discussion about students' individual assessments; (3) more time and privacy for students' assessment
	Focus group discussion and individual interviews				1 of 4 sub‐themes was not supported by illustration: (4) *difficulties in interpretation of specific concepts of a relevant assessment tool (no illustration)*
Carlsson et al. (2011) Sweden	Ethnography		The clinical training ward (CTW) at a university hospital was on a medical ward. Students worked and learned together as a team and were mentored by a team of mentors, thus receiving profession‐specific and team‐oriented mentoring	To describe how nurse mentors act when mentoring interprofessional student teams at a clinical training ward	Four major themes were identified: (1) supporting teamwork, (2) facilitating professional understanding, (3) breaking down barriers and (4) using a reflective approach
	Focus group and individual interviews				
	Observation				
	The constant comparative method				
Chen et al. (2016) United States	A qualitative description	13 mentors (10 physicians, 3 advanced practical nurses)	In three outpatient clinics, advanced practice nursing (APN) and medical students worked in teams by providing direct patient care. APNs and physicians mentored the student teams	To identify methods and supporting or impeding factors which mentors use to teach students from other professions in the clinical practice	Three major themes were identified: (1) a variety of teaching approaches and levels of engagement with trainees of different professions; (2) preceptor knowledge gaps related to curricula, goals and scope of practice of trainees from other professions and communications style; (3) administrative, structural and logistical elements that impact the success of precepting trainees from different professions in the clinical setting
	Individual interviews in person or via phone				
	Structured observation				
	Thematic analysis				
Chipchase et al. (2012) Australia	An exploratory case study	4 mentors (2 physiotherapists, 1 occupational therapist, 1 speech pathologist) 8 students (2 medicine, 2 physiotherapy, 2 occupational therapy, 2 speech pathology)	In Vietnam, in orphanages and schools for children with disabilities, students collaborated in the planning and delivery of programmes to improve children's health and assist the development of public health and health promotion programmes. The students were mentored by physiotherapists, occupational therapists and speech pathologists, who worked with students in different weeks. The medical mentor worked at the local hospital and was available if needed	Medical and allied health students and mentors' perceptions of the characteristics of mentoring in an interprofessional clinical practice	Mentors described that mentoring in an interprofessional clinical practice required changing the mentoring style in the interprofessional context, setting up regular briefing sessions, balancing learning directly from the clinical educator while asking questions to confirm students' approach and clear expectations of the students' professional contribution and a better understanding of their roles. Mentors also felt uncomfortable mentoring other professions and had difficulties with interprofessional mentoring and underlining the need for profession‐specific mentoring
	Focus group interviews and individual interviews before and after the clinical practice				
	Thematic analysis				
Conte et al. (2016) Sweden	A phenomenon	9 mentors (6 nurses, 3 physicians, 2 head mentors nurses) 8 students (3 medicine, 5 nursing)	An interprofessional education (IPE) unit was implemented in the intensive care unit, and medical and nursing students performed their clinical practice as an interprofessional team. One physician and one nurse mentored the team. A head mentor (a nurse) facilitated the interprofessional activities focusing on meta‐supervision	To describe issues that facilitate collaboration in teams of students in an IPE unit	Mentors described that they had facilitated students' collaboration by directing the strategies to expand the teams' reasoning, using time to support the team, balancing the students' contributions in their shared reasoning and facilitating common time together for the students. Mentors supported the teams in making joint decisions, creating a safe environment, guiding the students through reflection, keeping the team motivated and maintaining common motivation
	Semi‐structured interviews				
	Qualitative content analysis				
Dickie et al. (2019) Australia	Exploratory pilot study	4 mentors (2 nurses, 1 pharmacist, 1 social worker)	Interprofessional Clinical Supervision (IPCS) model implemented in acute care settings at Master Health. Students from medicine, nursing, pharmacy and allied health attended structured learning activities conducted by mentors. Mentors also conducted student rounding, providing face‐to‐face clinical support	To describe mentors' experiences during an interprofessional clinical placement	Three major themes were identified: (1) interprofessional clinical supervisor model development, (2) stakeholders' engagement and (3) interprofessional limitations
	Semi‐structured group interview				
	Thematic analysis				
Dyar et al. (2019) Sweden	Ethnography	21 mentors (7 nurses, 6 physicians, 3 healthcare assistants, 5 other staff) 19 students (12 nursing, 3 medicine, 4 healthcare assistants)	The student ward was placed on an acute medical ward in a teaching hospital. A pair of student nurses and one mentor cared for the Patients on the ward and medical students and healthcare assistant students were also present	To describe the characteristics of a learning environment in the student ward	Four major themes of how mentors supported students learning on the student ward were identified: (1) supporting student‐led learning; (2) supporting students learning together; (3) mentors' own approach to learning; (4) creating student‐dedicated space
	Observational study				
	Thematic analysis				
Ericson et al. (2017) Sweden	Mixed method study	**Interview:** 7 mentors (2 nurses, 2 physicians, 3 physiotherapists) 7 students (2 nursing, 4 medicine, 1 physiotherapist) 2 heads **Questionnaires:** 59 mentors (23 nurses, 31 physicians, 5 physiotherapists) 101 students (42 medicine, 27 nursing, 12 physiotherapy)	The Clinical Educational Department (KUM) was implemented in the emergency department for adult patients at the University Hospital. Student teams took care of patients with varying acute complaints. Students were mentored by mentors from each profession to develop interprofessional collaboration	To identify factors that facilitated or hindered the successful implementation of interprofessional clinical practice for students	Mentors found the allocated time for teaching stimulating to mentor committed students. They had to promote interprofessional collaboration among the students, build a team of mentors and *serve as role models for students to learn them to work together as a team (no illustration). The lack of continuity in the mentoring team due to scheduling difficulties proved to be a major concern for all mentors (no illustration)*
	Individual semi‐structured interviews				
	Questionnaires (included closed and open‐ended questions)				
	Observations				
	Qualitative data were analysed by using inductive thematic analysis. No mention on analysis of quantitative data				
Freeth et al. (2001) United Kingdom	Multi‐method methodology	**Interviews:** 10 mentors (all nurses) 13 clinical staffs 36 students (12 medicine, 12 nursing, 6 occupational therapy, 6 physiotherapy) **Questionnaires:** 36 students (12 medicine, 12 nursing, 6 occupational therapy, 6 physiotherapy) 34 patients	An interprofessional training ward for medical, nursing, occupational therapy and physiotherapy students was implemented in the rheumatology and orthopaedic ward at the Royal London Hospital. Students worked as an interprofessional team. The nurse mentors worked with an interprofessional student team. Nurse mentors provided profession‐specific mentoring for the nursing students. The other students in the team received profession‐specific mentoring from appropriate practitioners	To evaluate the interprofessional training ward	Mentors supported students learning on the training ward by providing students opportunities to work together. They had met initial difficulties mentoring their student groups and *implementing problem‐based learning within the constraints of delivering real‐time patient care (no illustration). They described not receiving advice or feedback about their mentoring (no illustration). Mentors described demanding concurrent responsibility for team mentoring and professional/individual mentoring (no illustration) and that each mentor has a particular mentoring style with their student teams (no illustration). Mentors considered the team reflection sessions to be immensely important in providing excellent learning opportunities (no illustration)*
	Semi‐structured interviews				
	Group interview				
	Questionnaires				
	Observations				
	Qualitative data were analysed by using thematic analysis. No mention on analysis of quantitative data				
Grace et al. (2016) Australia	Mixed methods	6 mentors (2 nurses, 1 osteopath, 3 naturopathy therapists) 16 students (naturopathy, nursing, osteopathy and pharmacy)	The online multi‐disciplinary resource aimed to prepare health students and their mentors for interprofessional clinical practice. The resource was trialled in four public and primary healthcare organizations with participants from naturopathy, nursing, osteopathy and pharmacy	To evaluate multi‐disciplinary resource	Mentors experienced that the online resource, which aimed to prepare students and mentors for interprofessional practice, supports them (1) to identify commonalities and respect differences and (2) to learn from each other through cross‐disciplinary communication and interaction
	Focus group interviews				
	Verbal and written feedback				
	E‐survey (closed and open‐ended questions)				
	Qualitative data were analysed by using thematic analysis				
	Quantitative data were analysed using the summary of survey responses function				
Jackman et al. (2016) Canada	Grounded theory	5 mentors (3 nurses, 2 physicians) 7 students (3 nursing, 4 medicine) 1 faculty coordinator managers (no mention of numbers)	Three nursing students and four medical students undertook interprofessional clinical practice in a rural acute care setting. Nurses and physicians are assigned to mentor the students	To explore mentors' and students' experiences of a process of the clinical practice and the factors that promoted or hindered the success of it	Mentors' point of view is that successful rural interprofessional clinical practice requires mentors to buy in, find opportunities to set up the students to discover interprofessional principles and support students' initiative. Hindrances to success were lack of logistical support and regret for lost interprofessional learning opportunities due to scheduling difficulties
	Semi‐structured individual and focus group interviews				
	Glaserian grounded theory				
Jacobsen et al. (2009) Denmark	A qualitative approach	3 mentors (1 head nurse, 1 occupational therapist, 1 physiotherapist) 8 students (2 nursing, 2 occupational therapy, 2 physiotherapy, 2 medicine) 1 assistant professor 1 project manager 1 researcher 3 heads	The Interprofessional Training Unit (ITU) was implemented in the orthopaedic ward. Students work as a team. Each profession has its clinical mentor at its disposal. The clinical mentors form a team that assumes patient responsibility and mentors students	To investigate the fulfilment of the goals of the ITU (ITU)	Mentors experienced that the success of interprofessional mentoring required setting patients' needs on the agenda for what the students will work with during the day and getting the students to teach each other. They underlined the time for discussion, evaluation and correction of teaching methods. As a part of the teaching, there is an expectation from the mentors that the students are in front and take professional responsibility. *Mentors were role models for students (no illustration) and were aware of the importance of being open‐minded and reflective (no illustration). Mentors gave the responsibility, emphasized seeing tasks from students' own professionals and created a secure environment with authentic tasks (no illustration)*
	Semi‐structured focus group and individual interviews				
	Systematic text condensation				
Jakobsen and Hansen (2014) Denmark	A qualitative case study	8 mentors (4 nurses, 2 physiotherapists, 2 occupational therapists) 17 students (no mention of professions) 2 clinical managers	The ITU was implemented in the orthopaedic wards. During a 2‐weeklong clinical practice, students worked as an interprofessional team and, under mentoring from trained interprofessional staff, provided care and rehabilitation to patients admitted for planned hip or knee arthroplasty	To explore the students' and the mentors' experiences of ITU concerning goal achievement including their emotions and beliefs	1 of 5 findings was supported by the illustration: (1) Something other than the expected happened
	Semi‐structured focus group interviews				*Not‐supported findings (1) the importance of getting to know each other (no illustration), (2) participating on equal footing (no illustration), (3) types of patients and possibilities for learning (no illustration), (4) logistic challenges and support (no illustration)*
	The inductive approach (Editing Organizing Style)				
Jakobsen et al. (2017) Denmark	An exploratory case study	5 mentors (2 nurses, 3 surgeons) 17 students, *n* = 17 (4 nursing, 13 medicine) 1 charge nurse	Interprofessional clinical practice was implemented in the orthopaedic outpatient clinic. Students examined and treated patients in collaboration. The nurse mentor who mentored the nursing students had mentoring as her main task. The surgeon who mentored the medical students most often simultaneously treated his patients in the outpatient clinic or the operation theatre	To explore how the interprofessional learning experience implemented and supported by mentors and managers	Mentors felt it was a challenging task to stand back to enable the students to learn, but they were also surprised because the students saw things that the mentors did not know about. They realized the necessity of reflecting and helped the students to observe, analyse and reflect. Mentors underlined the importance of providing interprofessional pre‐consultation instruction with both of the students and both of the mentors present. Mentors felt the managers' decisions and support were significant. They supported the task‐based approach in which the students were alone with the patients
	Semi‐structure interviews				
	Systematic text condensation				
Jensen et al. (2023) Norway	A social constructionist perspective and collective case study	**Interviews:** 4 mentors (no mentions of professions) 16 students (no mentions of professions) **Observation:** 19 mentors 47 students 6 patients	The study contexts were in a Norwegian community health centre, a Norwegian rehabilitation facility and a Swedish interprofessional training ward. In the first two contexts, the mentors were clinicians responsible for mentoring students on their professional and interprofessional teams. In the third context, the supervisor was a health professional with no clinical position, but an executive function for students in clinical placements	To explore how supervision facilitates and supports students' learning of patient centeredness in interprofessional clinical placements	Three major themes were identified: (1) alternating roles, presence and positioning; (2) illuminating interprofessional learning opportunities; (3) facilitating trust and independence
	Focus group interviews for mentors and students				
	Observation (in all three contexts)				
	a five‐step reflexive thematic analysis				
Lait et al. (2011) Canada	Not described	52 mentors (no mention of professions) 34 students (no mention of professions)	Interprofessional mentoring was implemented in a rural inpatient rehabilitation unit, a geriatric day centre, a geriatric centre, a respiratory department in an urban hospital and an acute and community care department in a rural hospital. Pharmacy, occupational therapy, physiotherapy, respiratory therapy, speech–language pathology and nursing students had official mentors responsible for teaching discipline‐specific skills and formal evaluation. In addition, students had other providers who acted as interprofessional mentors	To gather students' and interprofessional mentors' perceptions on interprofessional mentoring	Mentors felt that moving towards interprofessional mentoring requires all health professionals to contribute to students' learning by acting as mentors and to centre the students' learning around a ‘multi‐disciplinary patient’. Part of being an interprofessional mentor was to play a ‘coordinating role’ and to integrate students as members of the care team. *Mentoring activities consisted of observation and discussion (no illustration)*
	Group and individual semi‐structured interviews				
	Data were examined and coded for themes				
Martin et al. (2022) Australia	Qualitative action research	24 mentors (3 dietetics, 6 occupational therapy, 8 physiotherapy, 7 speech pathology) 34 students (6 dietetics, 6 occupational therapy, 9 physiotherapy, 13 speech pathology)	The Rural IPE and Supervision (RIPES) model was implemented across four rural health services. Students from dietetics, occupational therapy, physiotherapy and speech pathology undertook concurrent placements at RIPES sites. Local clinical mentors facilitated weekly tutorials, work shadowing and joint client sessions	To explore the enablers of and barriers to the implementation of the RIPES model	Main categories were identified: the value of the RIPES placement model, unintended benefits to the mentors, work units and rural areas, the tension between uniprofessional and IPE components and sustainability considerations
	Focus group interviews separately with students and mentors				
	Qualitative content analysis				
Missen et al. (2012) Australia	Mixed method	57 mentors (8 medicine, 16 nursing, 3 speech therapy, 3 occupational therapy, 3 social work, 2 dietetics, 5 physiotherapies, 1 psychologist, 4 pharmacies, 2 radiology, 3 dentistry, 3 podiatry, 2 medical imaging, 2 pathologies)	IPE in the clinical practice for healthcare students in rural acute health care	To obtain the views of healthcare professionals about the use of IPE in clinical education	The main results indicated that leadership was crucial to enable IPE to be implemented and sustained within the clinical learning environment and appropriate human and fiscal resources needed to be allocated. It was also found that participants saw current regulations promulgated by regulatory authorities and professional bodies to restrict opportunities for interprofessional mentoring of students within the healthcare setting
	Individual interviews				
	Focus group discussion				
	Semi‐structured questionnaire (open‐ended)				
	Thematic analysis				
Naumann et al. (2020) Australia	Social constructivist	5 mentors (2 nurse midwives, 1 speech pathologist, 1 nurse educator, 1 nursing director educator) 7 students (no mention of professions)	The interprofessional placement programme was implemented in six public hospital and health service organizations. The IPE programme consisted of a structured series of learning activities. Students from six health professions (nursing, psychology, physiotherapy, occupational therapy, speech pathology, nutrition and dietetics) were mentored by interprofessional mentors, who oversaw the clinical education activities of students undertaking placement	To explore the reflections of students and mentors in response to participation in a structured IPE clinical placement programme	Six major themes were identified: (1) mentors valued IPE, (2) preparation for interprofessional clinical placement needs to be embedded in curricula, (4) coordination and communication pathways need to be clear for both staff and students, (5) mentors recommended a continuation of IPE and (6) the value of IPE for students and patients
	Semi‐structured focus group				
	Inductive thematic analysis				
Oosterbaad‐Lodder et al. (2023) The Netherlands	A realist approach	12 mentors (2 nurses, 4 obstetric nurses, 3 midwives, 3 midwives + PA)	An ITU was implemented in the maternity ward of a teaching hospital. In this unit, a team of undergraduate healthcare students were responsible for planning, delivering and evaluating the treatments of mothers and their newborns under the mentoring of nursing and midwifery mentors	To identify the types of motivation of mentors, the factors that influence their motivation and how these factors can be used to facilitate the implementation and sustenance of ITUs	Factors that influenced the motivation for mentor's role mentoring in ITU were classified into four themes: (1) feeling appreciated for their role; (2) learning from others; (3) perceived effectiveness of guidance; (4) conflict of interest between guiding students and patient care. Within each of these themes were described 18 barriers and enablers, of which 5 were supported by illustration
	Interview				
	Directed content analysis to validate or conceptually the self‐determination theory				
Reeves and Freeth (2002) United Kingdom	Mixed method	10 mentors (no mention of professions) 36 students (12 nursing, 12 medicine, 6 occupational therapy, 6 physiotherapies) 34 patients (training ward) 34 patients (non‐training ward)	The ITU was implemented in the orthopaedic and rheumatology ward. Students worked in interprofessional teams. Each team was supported by a nurse mentor who worked closely with the team during their placement. In addition, all students received profession‐specific mentoring	To evaluate an interprofessional training ward pilot project	Mentors felt that providing continual facilitation for the student teams was ‘mentally straining’. *They were positive about the prospect of working on the interprofessional ward (no illustration), but felt not sufficiently prepared for the demands of the pilot ward (no illustration). Working on the training ward would contribute to mentors' personal, professional and academic development (no illustration). They provided a learning environment where the students could work more autonomously (no illustration), produced greater consistency in student team facilitation (no illustration) and greater alignment with the aims and objectives of the steering group (no illustration). Although the students were encouraged to work closely in their teams, the mentors from each profession tended to work in parallel, with little interprofessional collaboration (no illustration)*
	Individual interviews with the mentors				
	Group interviews with the students				
	Observations				
	Questionnaires with patients and students				
	Documentary evidence relating to the initiative				
	The analysis involved an initial search for recurring themes within each data set				
Reeves et al. (2002) United Kingdom	Mixed methods	8 mentors (no mention of professions) 36 students (12 nursing, 12 medicine, 6 occupational therapy, 6 physiotherapy) 11 clinical staffs 23 patients	The ITU was implemented in the orthopaedic and rheumatology ward. Students worked in interprofessional teams. Each team was supported by a nurse mentor who worked closely with the team during their placement. In addition, all students received profession‐specific mentoring	To obtain a comprehensive understanding of the impact of the ITU	The learning ward deepened mentors' facilitation skills and broadened their understanding of interprofessional collaboration. Mentors encouraged students to be autonomous and offered students more input. Mentors felt problems managing the demands of their ‘normal’ role and supporting the students on the training ward
	Semi‐structured interviews with mentors and clinical staff				
	Observations				
	Questionnaires with students and patients				
	Group interviews with the students				
	The analysis involved an initial search for recurrent themes				
Skinner, Robson, and Vien (2021) Australia	A complex theory framework	6 mentors (1 occupational therapist, 2 physiotherapists, 1 podiatrist, 2 speech pathologists)	The 5‐week Vietnam Programme provides an international and interprofessional clinical practice experience for occupational therapy, physiotherapy, podiatry and speech pathology students in an orphanage of children with disabilities. Students worked in interprofessional teams and were onsite and distance mentored by a team of Australian practitioners from each profession. A generic interprofessional assessment tool was developed for this programme, incorporating common core skills required of all students	To explore strengths, challenges and outcomes of the interprofessional assessment tool that was used on students' international and interprofessional clinical practice from the perspective of the mentors	Three major themes were identified: (1) interprofessional assessment tools supported acknowledging the student journey; (2) interprofessional assessment tools supported interprofessional authenticity; (3) interprofessional assessment tools supported collective and collaborative learning
	The semi‐structured focus group				
	Inductive thematic analysis				
Skinner, Simson, et al. (2021) Australia	Phenomenology	9 mentors (physiotherapy, occupational therapy, social work and dietetics)	Interprofessional work integrated learning (WIL), where mentors mentor students from other disciplines in rural and regional healthcare settings	To explore mentors' perceptions of interprofessional mentoring and assessment in interprofessional work‐integrated learning (WIL)	Three major themes were identified: (1) introducing the interprofessional lens early; (2) tapping into unique possibilities; (3) setting up for success – balancing challenges with opportunities
	Semi‐structured focus groups				
	A phenomenology of practice framework was used to interpret the data				
Toth‐Pal et al. (2020) Sweden	Mixed method	22 mentors and adjunct clinical lecturers (7 physiotherapists, 6 district nurses, 5 general practitioners, 2 occupational therapists, 1 dietician, 1 speech therapist) 109 students (39 nursing, 34 medicine, 19 physiotherapy, 11 occupational therapy, 4 speech therapy, 1 dietician, *n* = 1) 29 patients	Interprofessional home visits were organized during medical, nursing, physiotherapy, occupational therapy, speech therapy and dietician students' clinical practice in primary health care. Three or four students formed an interprofessional team and performed one home visit. A mentor accompanied them during the visit, but the students were responsible for history taking and performing relevant examinations	To evaluate person‐centred home visits as an educational model of interprofessional learning during healthcare students' clinical practice	15 of 17 sub‐categories of mentors' experiences supported by illustration were identified: (1) student activating in an effective way, (2) the mentor was a facilitator, which can be a new role for some mentors, (3) the students developed both in their professional and in their interprofessional roles, (4) the mentors experienced the activity as instructive and beneficial for patient care, (5) interprofessional assessments led to quality improvement for the patient, (6) difficult for mentors to allocate time for the seminars, (7) organize the activity required extensive planning, but there were tricks to facilitate it, (8) finding the right patient was a key factor, but it turned out to be difficult, (9) designating sufficient time for organizing the activity was perceived as a prerequisite, (10) separate organizations and geographical distances made cooperation more difficult, (11) the financial compensation for mentoring was less than the reimbursement for care visits, (12) support from the local management could make a big difference, (13) more engagement from and better co‐planning with health education programmes would facilitate IPL, (14) the project has opened for increased interprofessional collaboration in some cases, (15) support from the adjunct clinical lecturers was important. *Sub‐categories not supported by illustration: (1) more engagement from and better co‐planning with health education programmes would facilitate interprofessional learning, (2) experience of interprofessional collaboration in the present clinical work varied*
	Semi‐structured focus groups or individual interviews with mentors and adjunct clinical lecturers				
	The questionnaire with students and patients				
	Interview data were analysed using content analysis and questionnaires using descriptive statistics				

### 4.4 Data analysis

Qualitative research findings were pooled using JBI SUMARI with the meta‐aggregation approach (Lockwood et al., 2020). All findings from the primary studies that met the study's aim were extracted. A finding was defined as a verbatim extract of the authors' analytical interpretation. All extracted findings were classified as either unequivocal (U), if an illustration (i.e. quote of the participant, fieldwork observation or other data) was beyond a reasonable doubt; credible (C), if an illustration had no apparent association or NS, if an illustration did not support findings. Findings without an illustration were classified as NS. Only U and C findings were included in the meta‐aggregation. Firstly, U and C findings with similar meanings and themes were labelled and synthesized into categories. Secondly, defined categories with similar content were combined and developed into synthesized findings based on the consensus of all reviewers. The overall ConQual score for each synthesized finding was labelled as high, moderate, low or very low (Lockwood et al., 2020). All synthesized findings started with a high ranking due to the inclusion of only qualitative or qualitative share of mixed methods study. The ConQual score of the synthesized finding was downgraded to one level for each synthesized finding, with findings from studies with moderate dependability ratings and a mix of U and C findings (Table 3).

**TABLE 3:** ConQual summary of findings (Lockwood et al., 2020).

Synthesized finding	Type of research	Dependability	Credibility	ConQual score
Competences related to the preparation and development of interprofessional clinical practice	Qualitative study or mixed methods study^a^	Downgrade one level^b^	Downgrade one level^c^	Low
Competences relate to supporting the learning process in interprofessional clinical practice	Qualitative study or mixed methods study^a^	Downgrade one level^b^	Downgrade one level^c^	Low
Competences related to creating interprofessional mentor identity	Qualitative study or mixed methods study^a^	Downgrade one level^b^	Downgrade one level^c^	Low

^a^ Each synthesized finding started with a high ranking for qualitative or mixed methods studies.

^b^ Downgraded one level due to common dependability issues across the included studies (the majority of studies had no statement locating the researcher and no acknowledgement of their influence on the research).

^c^ Downgraded one level due to a mix of unequivocal/credible findings.

## 5 RESULTS

### 5.1 Characteristics of the included studies

The studies included in the review were published between 2001 and 2023 and were conducted across eight countries: Australia (8), Canada (2), Denmark (3), the Netherlands (1), Norway (1), Sweden (6), the United Kingdom (3) and the United States (1). A total of 314 mentors from 26 different background professions were involved in the studies. The most common professional groups were nursing (*n* = 73), medicine (*n* = 53) and allied health professions (*n* = 43). The studies were conducted in university hospitals, hospitals, outpatient clinics, geriatric centres and rural, primary, public, community and private healthcare settings. Seven of the included studies used mixed methods in design, and the others were qualitative. The studies used a variety of qualitative methodological approaches, including ethnography, phenomenology, grounded theory, social constructivist, realist and complex theory framework approaches, as well as exploratory pilot, case, qualitative descriptive and action research.

### 5.2 Review findings

**FIGURE 1:**
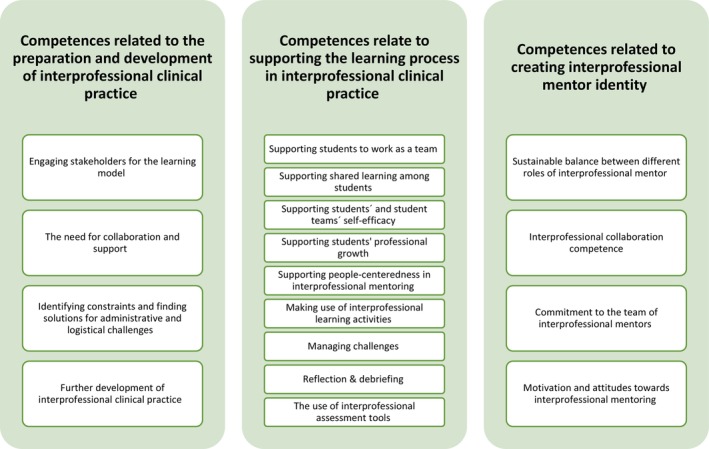
Healthcare professionals' competence in mentoring interprofessional students in clinical practice.

Three synthesized findings were generated from 17 categories (Figure 1). The categories comprised 109 findings, of which 102 were U and 7 were C (Appendix 3). The first synthesized finding included 4 categories reflected by 30 findings, the second included 9 categories reflected by 47 findings and the third included 4 categories reflected by 32 findings.

#### 5.2.1 Competences related to the preparation and development of interprofessional clinical practice

*Engaging participants in the learning model* included mentors' experiences of preparing students, mentors and all other stakeholders for the interprofessional clinical practice model and ensuring that clinical placements are structured and well organized. Mentors described that the move towards interprofessional clinical practice requires healthcare professionals to contribute to students' learning by acting as mentors (Lait et al., 2011). However, mentors experienced initial difficulties with interprofessional mentoring (Chipchase et al., 2012; Freeth et al., 2001) and felt uncomfortable mentoring other professionals (Chipchase et al., 2012). The role of interprofessional mentor may also be a new role for some mentors (Toth‐Pal et al., 2020). In this context, mentors felt it was essential to ensure that both students and mentors need to be engaged (Dickie et al., 2019), all participants understood the model and its benefits (Skinner, Simson, et al., 2021) and that coordination and communication were precise to participants beforehand (Naumann et al., 2020). To engage all stakeholders, mentors also emphasized the need to ensure that the placements were well organized and structured (Skinner, Simson, et al., 2021) and that the preparation for interprofessional clinical practice was included in the curriculum (Naumann et al., 2020).

*Identifying the need for collaboration and support* included mentors' experiences identifying the need for cooperation and support from managers, education providers, organizations and coordinators in preparing and developing interprofessional clinical practice. Mentors expressed the importance of managers' support (Jacobsen et al., 2009; Jakobsen et al., 2017; Missen et al., 2012; Toth‐Pal et al., 2020) and collaboration with education providers (Missen et al., 2012; Toth‐Pal et al., 2020) as crucial for the success of interprofessional mentoring. Mentors also experienced that a trained and resourced coordinator and smooth cooperation with the coordinator supported the implementation of IPE in healthcare settings (Missen et al., 2012).

*Identifying constraints and finding solutions for administrative and logistical challenges* included mentors' experiences of recognizing existing rules on professional boundaries that restrict interprofessional clinical practice and finding solutions for administrative and logistical challenges. Mentors had identified rules and guidelines on professional boundaries (Missen et al., 2012), scheduling difficulties (Grace et al., 2016), administrative, structural (Chen et al., 2016; Lait et al., 2011) and logistical elements (Chen et al., 2016; Grace et al., 2016; Jackman et al., 2016; Lait et al., 2011; Toth‐Pal et al., 2020) as well as university learning, restricts (Missen et al., 2012), which limited interprofessional clinical practice and required solutions to be found in order to implement and develop successful interprofessional clinical practice.

Nurses are often buddied with [to supervise] a medical student. However, nurses are currently not permitted to sign off skills objectives for medical students. (Missen et al., 2012; Medicine, 3a)

*Further development of interprofessional clinical practice* included mentors' experiences of being able to be involved in developing and streamlining interprofessional clinical practice. Mentors described that though they considered interprofessional clinical practice to be an excellent model, they also identified various areas for improvement to develop and streamline it, such as simplifying the learning activities (Toth‐Pal et al., 2020), conditions to make implementation more practical or appropriate for students or more accessible for mentors to share the workload (Martin et al., 2022) or more coordinated to ensure continuity (Naumann et al., 2020). In addition, mentors had experienced that interprofessional clinical practice could involve more junior students by tailoring the model to their knowledge and skills (Skinner, Simson, et al., 2021).

#### 5.2.2 Competences relate to supporting the learning process in interprofessional clinical practice

*Supporting students to work as a team* included mentors' experiences of supporting students to work as a team and integrate them as care team members. Mentors described that they need to promote interprofessional collaboration between the students (Carlsson et al., 2011; Ericson et al., 2017), support student teams to find structure in their collaboration, for example, by encouraging students to use written documents and think out loud (Conte et al., 2016; Freeth et al., 2001). Mentors have allowed students to work closely together (Freeth et al., 2001) and integrate students as care team members (Lait et al., 2011).

*Supporting shared learning among students* included mentors' experiences of supporting students to learn together and getting them to teach each other. It refers to how mentors support students in learning together and teaching each other (Dyar et al., 2019; Jacobsen et al., 2009). Mentors described how they had balanced students' contributions to shared reasoning and learning, for example, by refocusing the team's dialogue, redirecting questions and stressing the importance of exploring professional boundaries (Conte et al., 2016).

*Supporting the self‐efficacy of students and student teams* included mentors' experiences of being able to stand back and encourage student teams' autonomy and initiative and to make joint decisions. Mentors described it as very important but challenging to support students learning in interprofessional clinical practice by standing back (Jakobsen, 2016). They expressed that they needed to be able to take responsibility (Toth‐Pal et al., 2020), but at the same time to encourage student teams to be independent, take the initiative and make joint decisions with little mentoring (Grace et al., 2016; Jensen et al., 2023; Reeves et al., 2002). Mentors used various strategies to extend the teams' reasoning by balancing listening with encouragement and asking open questions (Conte et al., 2016). Self‐efficacy requires student‐led learning, where the role of the mentor is seen more as a guide, role model or helper rather than a teacher (Dyar et al., 2019).

I think that perhaps I'm sometimes a little too quick to give them the answer – so it's definitely something I could improve. (Jakobsen et al., 2017, p. 616; Interview 8, Surgeon)

*Supporting students' professional growth* included mentors' experiences supporting student's professional and interprofessional skills and expertise. Mentors recognized that it is essential (Carlsson et al., 2011) and possible (Toth‐Pal et al., 2020) for students to develop an understanding of both their professional roles and the professional roles of other team members during an interprofessional clinical practice. Mentors also stressed the importance of implementing interprofessional clinical practice as early as possible in students' studies to understand better the healthcare system and the different roles of each health professional (Skinner, Simson, et al., 2021). To make this explicit to students, mentors had, for example, directed profession‐specific questions from a student to a fellow student from the same profession, hence facilitating (Carlsson et al., 2011). Mentors also experienced that it was important for students to be assigned a mentor from their profession to provide profession‐specific perspectives and mentoring (Chipchase et al., 2012). When mentoring students from another profession, they expressed concerns about determining the student's clinical knowledge level and the appropriateness of the student's contribution to patient care (Skinner, Simson, et al., 2021).

*Supporting people centeredness in interprofessional mentoring* included mentors' experiences of being able to mentor in a people‐centred way and identify eligible patients for interprofessional clinical practice. Mentors described that the learning of the student groups had to be people centred and focus on people centeredness in interprofessional clinical practice (Jacobsen et al., 2009; Lait et al., 2011). Based on this, mentors stressed that finding the right patient is a crucial factor, albeit often tricky (Toth‐Pal et al., 2020).

*Making use of interprofessional learning activities* included mentors' experiences of being able to use effective interprofessional learning opportunities and activities. Mentors needed to illuminate interprofessional learning opportunities for students, for example, by asking the right questions and guiding students to work in an interprofessional way (Jensen et al., 2023) and trying to find opportunities to set up the students to discover interprofessional principles themselves (Grace et al., 2016) by supporting the task‐based approach in which the students were alone with the patients (Jakobsen et al., 2017). Mentors also brought up how organizing these activities required extensive planning, but they also mentioned that the activity became smoother each time it was repeated (Toth‐Pal et al., 2020).

*Managing challenges* included mentors' experiences of managing various upcoming challenges. Mentors have faced and settled different challenges when mentoring interprofessional clinical practice. Despite the pre‐planning of the pedagogical approach and practical arrangements for interprofessional clinical practice, mentors also had to be prepared for something other than expected (Jakobsen & Hansen, 2014). They have recognized a tension between profession‐specific and interprofessional placement requirements due to time commitment and concerns about developing skills in students' professions (Martin et al., 2022) and also an imbalance between the complexity of the work and the experience level of the teams, when mentors were challenged to keep the student team motivated (Conte et al., 2016). Mentors have emphasized the importance of contributing to a breakdown of hierarchical barriers by making the different knowledge domains and professional responsibilities visible and understandable to students and focusing on people centeredness as a basis for team decision making on patient care (Carlsson et al., 2011).

*Reflection and debriefing* included mentors' experiences of being able to organize and hold interprofessional reflection and debriefing sessions. When mentoring interprofessional clinical practice, mentors have experienced the value of regular reflection (Carlsson et al., 2011; Jakobsen, 2016) and debriefing (Chipchase et al., 2012) sessions for interprofessional student learning. Mentors described that it was essential to create a safe environment and guide the students through the reflection (Conte et al., 2016; Jakobsen, 2016). Mentors highlighted that the timing of these sessions was significant in relation to the specific situation being discussed (Conte et al., 2016) and that they focused on clinical and collaborative aspects (Chipchase et al., 2012).

We always debriefed as a group at lunch time and we always debriefed as a group after …. Even though that sounds quite excessive, that was absolutely necessary. (Chipchase et al., 2012, p. 468; Zoe (mentor))

*Interprofessional assessment tools* included mentors' experiences of being able to use interprofessional assessment tools for student evaluation. Mentors have experienced challenges in assessing students from another profession if the focus is on profession‐specific clinical reasoning, and therefore, particular attention should be paid to appropriate assessment in an interprofessional context (Skinner, Simson, et al., 2021). The use of interprofessional assessment tools has been experienced by mentors as a relevant working tool for student assessment, supporting recognition of the student journey, interprofessional authenticity and collective and collaborative learning (Skinner, Robson, & Vien, 2021) and stimulating discussion about individual student assessments (Andermo et al., 2023).

#### 5.2.3 Competences related to creating interprofessional mentor identity

*The sustainable balance between different roles of interprofessional mentors* included mentors' experiences of being able to allocate resources for interprofessional mentoring. Mentors described the continual mentoring of the students' teams was ‘mentally straining’ (Reeves & Freeth, 2002). They often had concurrent and overlapping mentoring and patient care responsibilities and had experienced it challenging to balance these roles (Oosterbaad‐Lodder et al., 2023; Reeves et al., 2002). When mentoring student teams, mentors also had to balance an alternating role, presence and positioning of mentors, being available and giving the student team sufficient responsibility to work independently without compromising patient safety (Jensen et al., 2023). Mentors emphasized that allocating human and fiscal resources (Missen et al., 2012), creating dedicated spaces for students (Dyar et al., 2019) and more time and privacy for student assessment (Andermo et al., 2023) were crucial factors in balancing different roles and mentoring success.

*Interprofessional collaboration competence* included mentors' experiences of knowing the meaning of interprofessional collaboration and required competencies. The transition from profession‐specific to interprofessional clinical practice may reveal mentors' interprofessional limitations, for example, related to professional biases and assumptions (Dickie et al., 2019). Mentors also identified interprofessional knowledge gaps related to curriculum, goals, scope of practice of students from other professions and communication styles (Chen et al., 2016). Mentors had experienced that online courses designed to prepare students and mentors for interprofessional practice strengthened their interprofessional competencies, such as identifying commonalities, respecting differences and learning from each other through interprofessional communication and interaction (Grace et al., 2016). Mentors also highlighted that mentoring in the interprofessional clinical practice had broadened mentors' understanding of interprofessional collaboration (Reeves et al., 2002), increased interprofessional collaboration in their practice and improved patient care (Martin et al., 2022; Toth‐Pal et al., 2020).

*Commitment to the team of interprofessional mentors* included mentors' experiences of adopting the role of interprofessional mentor and being committed to a mentoring team. Mentors described the need to change their mentoring style in the interprofessional context (Chipchase et al., 2012). This involved building a team of mentors (Ericson et al., 2017), the ability to develop a ‘team identity’ (Dickie et al., 2019) and enabling peer learning (Jakobsen et al., 2017). An important aspect of team mentoring is the mentors' shared commitment to the learning approach (Dyar et al., 2019), which includes getting to know the students and adapting the approach to their individual learning goals, previous experiences, strengths and weaknesses and learning styles. Mentors had various teaching approaches and engagement levels with students of different professions (Chen et al., 2016).

Because we were all part‐time, it was hard to know what each other was approaching the role. It would have been better to be working across more shifts together. (Dickie et al., 2019, p. 813; Supervisor 2)

*Motivation and attitudes towards interprofessional mentoring* included mentors' experiences of recognizing the value of IPE for students and patients and feeling appreciation for their role as an interprofessional mentor. Mentors value IPE (Martin et al., 2022; Naumann et al., 2020). They recognized its importance for students, patients (Martin et al., 2022; Naumann et al., 2020), patient care (Toth‐Pal et al., 2020) and the work unit (Martin et al., 2022). Mentors have also seen IPE as tapping into unique possibilities such as increased opportunities to expand placement and mentoring capacity, learning more about other professions and a pathway for junior mentors (Skinner, Simson, et al., 2021). Learning from others, perceiving the effectiveness of mentoring and feeling appreciated for the mentor role have increased mentors' motivation (Oosterbaad‐Lodder et al., 2023). They have found the time allocated to teaching stimulating for mentoring committed students (Ericson et al., 2017) and have been surprised by the competence of student teams (Jakobsen, 2016). On the other hand, mentors experienced that the financial compensation for mentoring was low for the task's demands (Toth‐Pal et al., 2020).

## 6 DISCUSSION

This meta‐aggregation provides a comprehensive overview of the integrated findings of qualitative studies exploring mentors' experiences of their competence in mentoring interprofessional clinical practice. The review's findings reveal that mentors need to be able to prepare and develop an interprofessional clinical placement that emphasizes participants' engagement in the learning model. Lie et al. (2016) have identified engagement as one of the critical challenges for the future of IPE to increase mentors' interest in, comfort with and competence in effectively mentoring interprofessional student teams. Engagement in interprofessional clinical practice is essential not only for mentors but also for students. Engaging students in the interprofessional clinical practice model has also been identified as a critical factor in ensuring a positive learning experience for students and the success of IPE efforts (Barr et al., 2017). Mentors can, for example, support students' preparation for interprofessional clinical practice through an induction session where students get to know each other, learn about IPE and interprofessional collaboration and jointly develop their communication skills (Mink et al., 2021).

As found in this review, profession‐specific mentoring also emphasized the competencies of mentors to develop collaboration with education providers, where the need for collaboration was related, especially in difficult mentoring situations and the need to clarify expectations between individuals and organizations (Tuomikoski et al., 2020). In this review, mentors recognized the need for collaboration with education providers in interprofessional clinical practice, particularly in co‐planning, curriculum development and finding solutions to administrative and logistical challenges. An effective connection between education providers and mentors is essential to ensuring a safe learning environment for students in interprofessional clinical practice (O'Brien et al., 2019). In addition, the pedagogical competence of education providers can be leveraged, especially when implementing new models of interprofessional clinical practice (O'Brien et al., 2019). This is particularly important when mentors are not required to have mentoring training (Mikkonen et al., 2022) or specific interprofessional mentoring training (Mattiazzi et al., 2024; Oosterom et al., 2019) and do not have sufficient competence in interprofessional collaboration and mentoring student teams (Kent et al., 2017). The continuity and sustainability of the interprofessional mentoring model should be considered from the beginning of the implementation of interprofessional clinical practice (Lim & Noble‐Jones, 2018). This requires the identification of needs for further development of mentoring and the readiness and willingness of mentors to develop their own mentoring and mentoring practice in general, as well as a commitment to longer term change, which competencies emerged from the mentors' experiences in this review.

The mentors' competence focuses on supporting the student's learning process, including pedagogical issues, feedback and student evaluation (Mikkonen et al., 2020; Tuomikoski et al., 2018, 2020). Although there are elements similar to those supporting the learning process in interprofessional mentoring as in individual mentoring, the results of this review also revealed some differences. The results of this review indicated that supporting the learning process included the competence to facilitate and support students to work as a team and share learning among students. Team facilitation requires understanding team dynamics and team performance (IPEC, 2023; Schmitz et al., 2017) and identifying and resolving team conflicts (Schmitz et al., 2017). This review found that mentors need to be able to support students in developing both their professional and interprofessional competencies and expertise during interprofessional clinical practice. In this regard, mentors expressed concern about interprofessional clinical practice to provide sufficient profession‐specific growth. A previous literature review shows that goals related to profession‐specific competencies, interprofessional learning and professional identity formation have been achieved through different pedagogical approaches in interprofessional clinical practice (Jakobsen, 2016). However, in interprofessional clinical practice, students' profession‐specific competencies, individual learning goals and competence development also need to be considered and enabled during interprofessional clinical practice.

Providing accurate and timely feedback to students on their progress towards achieving interprofessional competencies and learning outcomes is critical to health professional programmes. Feedback should be situational, private, objective, honest, positive, constructive, immediate, continuous, timely and based on the student's goals (Tuomikoski et al., 2020). None of the studies in this review highlighted the experiences of competence in providing feedback, but this interprofessional clinical mentoring culminated in reflection and debriefing. Competence in reflection and debriefing was seen as a two way; mentors had to create and lead reflection and debriefing situations and facilitate students in reflecting and acting as a team in debriefing situations. Reflection is seen as a formal process that can assist students in developing necessary competencies in clinical practice; for example, the choice of framework and mentoring strategies can develop students' reflective capacity and promote reflection (Scheel et al., 2021).

Evaluation of student performance is part of the student's clinical practice, for which mentors are responsible. Nursing mentors have rated their evaluation skills as their weakest area of mentoring competence (Tuomikoski et al., 2018), while students themselves perceive evaluation and being constantly evaluated as a particularly stressful factor in their practical training (Bhurtun et al., 2021). Interprofessional clinical practice is highly context specific and often subject to unpredictable variability. As such, the design and implementation of appropriate, fair and standardized assessment is recognized as a formidable undertaking (Almoghirah et al., 2021). In this review, the mentors found various tools valuable and supportive when evaluating students' interprofessional competencies. Mentors could use several assessment tools for evaluating students' interprofessional competence (Almoghirah et al., 2021) in interprofessional clinical practice. However, these tools should be clear enough, and mentors should have the necessary skills. When using these tools in interprofessional clinical practice, it is also possible to include peer assessment in the evaluation. This may also reduce the stress of the evaluation for students, as peer support is known to help some students overcome challenges (O'Brien et al., 2019).

This review found that mentors had to create an interprofessional mentor identity, which involved a sustainable balance between the different roles of an interprofessional mentor. Mentors in this review experienced challenges in balancing patient care and mentoring roles. This finding is consistent with the previous study (Tuomikoski et al., 2018). Mentors need to find an organizational balance between the different roles, affecting the mentor's motivation (Oosterbaad‐Lodder et al., 2023) and the student's learning experience (Kent et al., 2017).

This review also found that mentors need strong interprofessional competencies in interprofessional clinical practice, though some mentors had identified their interprofessional competencies as inadequate. In addition, mentors in interprofessional clinical practice face particular challenges in mentoring students from different backgrounds and at different stages of their programme. This requires mentors to be familiar with the core competencies and professional tasks of different professions (IPEC, 2023; Kent et al., 2017; Schmitz et al., 2017) and the goals and characteristics of different professional programmes (Kreider et al., 2022). Mentors may benefit from continuing education in interprofessional competencies (Kent et al., 2017).

In interprofessional clinical practice, mentors should support students in the learning process and other mentors (Barr et al., 2017). In this review, the mentors experienced the need to build a mentoring team with shared goals, a common approach to teaching and an understanding of interprofessional clinical practice. Co‐teaching with different professions requires a shared commitment to mentoring strategies, roles and learning activities, resolving interprofessional friction and resolving conflicts (Lie et al., 2016). In addition, interprofessional mentors should clearly understand their role in supporting students and know how their attitudes and behaviours affect group functioning (Botma & Labuschagne, 2019).

## 7 CONCLUSION

Many health profession programmes emphasize the importance of IPE by including it in health profession programme accreditation and requiring that student clinical practice promotes interprofessional collaborative practice (e.g. European Council, AAMCE, AACN). This requires healthcare organizations to integrate effective implementation of IPE into clinical practice. This systematic review provides insights into mentors' experiences of the specific competencies required for interprofessional mentoring. Competent interprofessional mentors are essential for implementing, developing and sustaining interprofessional clinical practice. Understanding mentors' experiences of interprofessional mentoring competencies is crucial as it informs what successful interprofessional clinical practice requires of mentors. It is essential to support the development of mentors' interprofessional mentoring competence and the collaboration of different professional groups and educational organizations in the development of student mentoring. Interprofessional mentoring requires specific competencies from mentors, and this should also be included in the content of the various mentoring educations.

## 8 LIMITATIONS AND STRENGTHS

This systematic review follows the JBI guidelines for qualitative synthesis and a transparent process. However, some limitations should be acknowledged. First, according to JBI guidelines, only findings supported by illustrations were included in the meta‐aggregation. In this review, 7 out of 25 included studies used a mixed methods approach, and 18 out of 25 studies included additional participants other than mentors (Table 2). For example, the individual research articles could not provide illustrations for all sub‐categories due to word count limitations. To gain a comprehensive overview of the mentors' experiences, we also analysed NS findings separately. These findings did not reveal new categories, but instead reinforced existing results of meta‐aggregation with U and C findings. NS findings have been presented separately and marked in italics in the extraction (Table 2) and Appendix 3 to ensure transparency. Secondly, many of the studies included in this review focused on experiences of interprofessional clinical practice, a new model of mentoring in organizations and new to mentors. Therefore, mentors may have described only first and short‐term experiences of mentoring competence in interprofessional clinical practice. However, these experiences are essential, especially when embedding the new mentoring model. More research on mentors' long‐term experiences and comprehensive quantitative research on mentors' competencies is needed. Thirdly, only studies published in English, Swedish or Finnish and peer‐reviewed studies were included, so relevant research may have been omitted. However, this review represents global research on the topic.

## AUTHOR CONTRIBUTIONS

All authors have agreed on the final version and meet at least one of the following criteria (recommended by the ICMJE*): (1) substantial contributions to conception and design, acquisition of data or analysis and interpretation of data; (2) drafting the article or revising it critically for important intellectual content. *http://www.icmje.org/recommendations/.

## FUNDING INFORMATION

This research received no specific grant from any funding agency in the public, commercial or not‐for‐profit sectors.

## CONFLICT OF INTEREST STATEMENT

No conflict of interest has been declared by the authors.

### PEER REVIEW

The peer review history for this article is available at https://www.webofscience.com/api/gateway/wos/peer‐review/10.1111/jan.16347.

## Supporting information


Appendix S1.

## Data Availability

No data are available.

## APPENDIX 1 THE COMPREHENSIVE SEARCH STRATEGY

### 1.1

**Table:**

Keywords	Cinahl	PubMed	Scopus	Medic	ProQuest
Population					
mentor OR supervisor OR facilitator OR preceptor OR coacher OR tutor OR trainer OR educator OR instructor OR clinical guide OR clinical instructor OR clinical teacher OR mentorship	(MH ‘Mentorship’) OR (MH ‘Clinical Supervision’) OR (MH ‘Supervisors and Supervision+’) OR (MH ‘Preceptorship’) OR (MH ‘Teaching Methods, Clinical’) OR mentor* OR supervisor* OR facilitator* OR preceptor* OR coacher* OR tutor* OR trainer* OR educator* OR instructor* OR ‘clinical guide*’ OR ‘clinical teacher*’ OR mentorship*	(‘Mentors’[Mesh]) OR (‘Nursing, Supervisory’[Mesh]) OR (‘Preceptorship’[Mesh]) OR (‘Teacher Training’[Mesh]) OR (‘Health Educators’[Mesh]) OR mentor*[Text Word] OR supervisor*[Text Word] OR facilitator*[Text Word] OR preceptor*[Text Word] OR coacher*[Text Word] OR tutor*[Text Word] OR trainer*[Text Word] OR educator*[Text Word] OR instructor*[Text Word] OR ‘clinical guide*’[Text Word] OR ‘clinical teacher*’[Text Word] OR mentorship*[Text Word]	TITLE‐ABS‐KEY (mentor* OR supervisor* OR facilitator* OR preceptor* OR coacher* OR tutor* OR trainer* OR educator* OR instructor* OR ‘clinical guide*’ OR ‘clinical teacher*’ OR mentorship*)		noft(mentor* OR supervisor* OR facilitator* OR preceptor* OR coacher* OR tutor* OR trainer* OR educator* OR instructor* OR ‘clinical guide’ OR ‘clinical teacher*’ OR mentorship*)
Exposer of interest					
interprofessional, multi‐professional, indisciplinary, interdiscipline, multi‐disciplinary, interdisciplinary, transdisciplinary, transprofessional	(MH ‘Education, Interdisciplinary’) OR interprofessional* OR inter‐professional* OR multiprofessional* OR multi‐professional* OR indisciplinar* OR in‐disciplinar* OR interdisciplin* OR inter‐disciplin* OR multidisciplinar* OR multi‐disciplinar* OR transdisciplinar* OR trans‐disciplinar* OR transprofessional* OR trans‐professional*	(‘Interprofessional Education’[Mesh]) OR (‘Interdisciplinary Placement’[Mesh]) OR interprofessional*[Text Word] OR inter‐professional*[Text Word] OR multiprofessional*[Text Word] OR multi‐professional*[Text Word] OR indisciplinar*[Text Word] OR in‐disciplinar*[Text Word] OR interdisciplin*[Text Word] OR inter‐disciplin*[Text Word] OR multidisciplinar*[Text Word] OR multi‐disciplinar*[Text Word] OR transdisciplinar*[Text Word] OR trans‐disciplinar*[Text Word] OR transprofessional*[Text Word] OR trans‐professional*[Text Word]	TITLE‐ABS‐KEY(interprofessional* OR inter‐professional* OR multiprofessional* OR multi‐professional* OR indisciplinar* OR in‐disciplinar* OR interdisciplin* OR inter‐disciplin* OR multidisciplinar* OR multi‐disciplinar* OR transdisciplinar* OR trans‐disciplinar* OR transprofessional* OR trans‐professional*)	moniammat* OR monialai* OR monitietei* OR ‘tieteiden välinen’ OR ‘tieteiden välisyys’	noft(interprofessional* OR inter‐professional* OR multiprofessional* OR multi‐professional* OR indisciplinar* OR in‐disciplinar* OR interdisciplin* OR inter‐disciplin* OR multidisciplinar* OR multi‐disciplinar* OR transdisciplinar* OR trans‐disciplinar* OR transprofessional* OR trans‐professional*)
Context					
learning environment clinical, education clinical, students placement, clinical practice, clinical rotation, clinical training, clinical learning, clinical teaching, clinical learning environment, internship, clinical education unit, clinical learning unit, team preceptorship, clinical teaching unit, collaborative learning unit, training unit, training ward	(MH ‘Learning Environment, Clinical’) OR (MH ‘Education, Clinical+’) OR (MH ‘Student Placement’) OR (MH ‘Clinical Competence+’) OR (MH ‘Teaching Methods, Clinical+’) OR (MH ‘Internship and Residency’) OR (MH ‘Interns and Residents’) OR ‘learning environment clinical*’ OR ‘education clinical*’ OR ‘student placement*’ OR ‘clinical practice*’ OR ‘clinical rotation*’ OR ‘clinical training*’ OR ‘clinical learning*’ OR ‘clinical teaching*’ OR ‘clinical learning environment*’ OR internship* OR ‘clinical placement*’ OR ‘clinical education*’ OR ‘clinical learning unit*’ OR ‘team preceptorship*’ OR ‘clinical teaching unit*’ OR ‘collaborative learning unit*’ OR ‘training unit*’ OR ‘training ward*’	(‘Preceptorship’[Mesh]) OR (‘Teaching Rounds’[Mesh]) OR (‘Internship and Residency’[Mesh]) OR (‘Internship, Nonmedical’[Mesh]) OR (‘learning environment clinical*’[Text Word]) OR (‘education clinical*’[Text Word]) OR (‘student placement*’[Text Word]) OR (‘clinical practice*’[Text Word]) OR (‘clinical rotation*’[Text Word]) OR (‘clinical training*’[Text Word]) OR (‘clinical learning*’[Text Word]) OR (‘clinical teaching*’[Text Word]) OR (‘clinical learning environment*’[Text Word]) OR (internship*[Text Word]) OR (‘clinical placement*’[Text Word]) OR (‘clinical education*’[Text Word]) OR (‘clinical learning unit*’[Text Word]) OR (‘team preceptorship*’[Text Word]) OR (‘clinical teaching unit*’[Text Word]) OR (‘collaborative learning unit*’[Text Word]) OR (‘training unit*’[Text Word]) OR (‘training ward*’[Text Word])	TITLE‐ABS‐KEY (‘learning environment clinical*’ OR ‘education clinical*’ OR ‘student placement*’ OR ‘clinical practice*’ OR ‘clinical rotation*’ OR ‘clinical training*’ OR ‘clinical learning*’ OR ‘clinical teaching*’ OR ‘clinical learning environment*’ OR internship* OR ‘clinical placement*’ OR ‘clinical education*’ OR ‘clinical learning unit*’ OR ‘team preceptorship*’ OR ‘clinical teaching unit*’ OR ‘collaborative learning unit*’ OR ‘training unit*’ OR ‘training ward*’)	‘Käytännön harjoittelu’ OR ‘kliininen harjoittelu’ OR työharjoit* OR harjoit* OR ‘moniammatillinen harjoittelu’ OR ‘moniammatillinen koulutus’ OR ‘monialainen harjoittelu’ OR opetusmod* OR opiskelijamod* OR opetusosas* OR opiskelijaosas*	noft(‘learning environment clinical*’ OR ‘education clinical*’ OR ‘student placement*’ OR ‘clinical practice*’ OR ‘clinical rotation*’ OR ‘clinical training*’ OR ‘clinical learning*’ OR ‘clinical teaching*’ OR ‘clinical learning environment*’ OR internship* OR ‘clinical placement*’ OR ‘clinical education*’ OR ‘clinical learning unit*’ OR ‘team preceptorship*’ OR ‘clinical teaching unit*’ OR ‘collaborative learning unit*’ OR ‘training unit*’ OR ‘training ward*’)

## APPENDIX 2 ASSESSMENT OF METHODOLOGICAL QUALITY OF INCLUDED STUDIES BASED ON JBI‐QARI (LOCKWOOD ET AL., 2020)

### 2.1

**Table:**

Citation	Q1	Q2	Q3	Q4	Q5	Q6	Q7	Q8	Q9	Q10	Score
Andermo et al. (2023)	N	Y	Y	Y	Y	N	N	Y	Y	Y	7
Carlsson et al. (2011)	Y	Y	Y	Y	Y	N	Y	Y	Y	Y	9
Chen et al. (2016)	Y	Y	Y	Y	Y	Y	Y	Y	U	Y	9
Chipchase et al. (2012)	N	Y	Y	Y	Y	Y	Y	Y	Y	Y	9
Conte et al. (2016)	Y	Y	Y	Y	Y	N	N	Y	Y	Y	8
Dickie et al. (2019)	N	Y	Y	Y	Y	N	N	Y	Y	Y	7
Dyar et al. (2019)	Y	Y	Y	Y	Y	Y	Y	Y	Y	Y	10
Ericson et al. (2017)	Y	Y	Y	Y	Y	Y	Y	Y	Y	Y	10
Freeth et al. (2001)	Y	Y	Y	Y	Y	N	N	Y	N	Y	7
Grace et al. (2016)	Y	Y	Y	Y	Y	N	N	Y	Y	Y	8
Jackman et al. (2016)	Y	Y	Y	Y	Y	N	N	Y	Y	Y	8
Jacobsen et al. (2009)	N	Y	Y	Y	Y	Y	Y	Y	N	Y	8
Jakobsen and Hansen (2014)	N	Y	Y	Y	Y	Y	Y	Y	Y	Y	9
Jakobsen et al. (2017)	N	Y	Y	Y	Y	N	N	Y	Y	Y	7
Jensen et al. (2023)	Y	Y	Y	Y	Y	Y	Y	Y	Y	Y	10
Lait et al. (2011)	N	Y	Y	Y	Y	N	N	Y	Y	Y	7
Martin et al. (2022)	N	Y	N	Y	Y	Y	N	Y	Y	Y	7
Missen et al. (2012)	Y	Y	Y	Y	Y	N	N	Y	Y	Y	8
Naumann et al. (2020)	Y	Y	Y	Y	Y	Y	N	Y	Y	Y	9
Oosterbaad‐Lodder et al. (2023)	Y	Y	Y	Y	Y	Y	Y	Y	Y	Y	10
Reeves and Freeth (2002)	U	Y	Y	Y	Y	Y	Y	Y	N	Y	8
Reeves et al. (2002)	U	Y	Y	Y	Y	Y	Y	Y	Y	Y	9
Skinner, Robson, and Vien (2021)	N	Y	Y	Y	Y	Y	N	Y	Y	Y	8
Skinner, Simson, et al. (2021)	Y	Y	Y	Y	Y	N	Y	Y	Y	Y	9
Toth‐Pal et al. (2020)	N	Y	Y	Y	Y	N	Y	Y	Y	Y	8
Total (%)	52	100	96	100	100	52	52	100	84	100	

*Note*: JBI critical appraisal checklist for qualitative research: Q1 = Is there congruity between the stated philosophical perspective and the research methodology? Q2 = Is there congruity between the research methodology and the research question or objectives? Q3 = Is there congruity between the research methodology and the methods used to collect data? Q4 = Is there congruity between the research methodology and the representation and analysis of data? Q5 = Is there congruity between the research methodology and the interpretation of results? Q6 = Is there a statement locating the researcher culturally or theoretically? Q7 = Is the influence of the researcher on the research, and vice‐ versa, addressed? Q8 = Are participants, and their voices, adequately represented? Q9 = Is the research ethical according to current criteria or, for recent studies, is there evidence of ethical approval by an appropriate body? Q10 = Do the conclusions drawn in the research report flow from the analysis, or interpretation, of the data?

Abbreviations: N, no; U, unclear; Y, yes.

## APPENDIX 3 SYNTHESIZED FINDINGS

### 3.1

**Table:**

Findings	Categories	Synthesized findings
**Unequivocal (U) and credible (C) findings:** Stakeholder engagement (Dickie et al., 2019) (C) Preparation for interprofessional placements needs to be embedded in curricula (Naumann et al., 2020) (U) The importance of a coordinated approach, with clear guidelines to ensure supervisors and students have a shared understanding (Skinner, Simson, et al., 2021) (C) Ensure placements were well organized and structured (Skinner, Simson, et al., 2021) (U) Coordination and communication pathways need to be clear for staff and students (Naumann et al., 2020) (U) Moving towards interprofessional mentoring requires that all health professionals in the service area contribute to students' learning by acting as mentors (Lait et al., 2011) (U) The experience of initial difficulties mentoring their student groups (Freeth et al., 2001) (U) To feel uncomfortable supervising other professions (Chipchase et al., 2012) (U) To feel difficulties with interprofessional supervision (Chipchase et al., 2012) (U) The supervisor was a facilitator, which can be a new role for some supervisors (Toth‐Pal et al., 2020) (U)	**Engaging participants in the learning model** Able to prepare students, mentors and all other stakeholders for the model of interprofessional clinical practice. Able to ensure that clinical placements are structured and well organized	Competencies related to the preparation and development of interprofessional clinical practice
***Not‐supported findings (NS), not included in meta‐aggregation:*** *The experience of mentors' difficulty with implementing PBL within the constraints of delivering real‐time patient care* (Freeth et al., 2001) *(NS)* *To produce greater alignment with the aims and objectives of the steering group* (Reeves & Freeth, 2002) *(NS)* *Mentors felt not to be sufficiently prepared for the demands of the pilot ward* (Reeves & Freeth, 2002) *(NS)*		
**Unequivocal (U) and credible (C) findings:** The significance of the managers' decision and support for the project (Jakobsen et al., 2017) (U) Leadership across the organization is crucial to the successful implementation of IPE (Missen et al., 2012) (U) Support from the local management could make a big difference (Toth‐Pal et al., 2020) (U) The heads have had the attitude that there should be time for discussion, evaluation and correction of teaching methods (Jacobsen et al., 2009) (U) Education providers' role in leading and facilitating opportunities of interprofessional education (Missen et al., 2012) (U) Support from the adjunct clinical lecturers was important (Toth‐Pal et al., 2020) (U) For interprofessional education to be a realistic organizational goal, a dedicated coordinator needs to be suitably trained and resourced to facilitate the process (Missen et al., 2012) (U) Interprofessional education needs drivers/champions to coordinate and implement IPE within the healthcare setting (Missen et al., 2012) (U)	**Identifying the need for collaboration and support** Able to identify the need for cooperation and support from managers, education providers, organizations and coordinators in preparing and developing interprofessional clinical practice	
***Not‐supported finding (NS), not included in meta‐aggregation:*** *More engagement from and better co‐planning with health education programmes would facilitate IPL* (Toth‐Pal et al., 2020) *(NS)*		
**Unequivocal (U) and credible (C) findings:** The current regulations and/or guidelines promulgated by regulatory authorities and professional bodies are seen to restrict opportunities for interprofessional supervision of students within the healthcare setting (Missen et al., 2012) (U) Regret for lost IPE opportunities due to scheduling difficulties (Grace et al., 2016) (U) Administrative, structural and logistical elements that impact the success of precepting trainees from different professions in the clinical setting (Chen et al., 2016) (U) The logistical challenge of coordinating students' schedules (Jackman et al., 2016) (U) Logistical challenges related to the rural context for the staff members, as expert generalists, were already spread thin (Grace et al., 2016) (U) ‘Conventional’ university learning restricts the exposure of students to opportunities for interprofessional education because discipline‐specific education and learning objectives take precedence (Missen et al., 2012) (U) Part of being a mentor was to play a ‘coordinating role’ by connecting students with staff across disciplines and to be receptive to having other staff members' students join them (Lait et al., 2011) (U) Separate organizations and geographical distances made cooperation more difficult (Toth‐Pal et al., 2020) (U)	**Identifying constraints and finding solutions for administrative and logistical challenges** Able to recognize existing rules and guidelines on professional boundaries restrict interprofessional clinical practice. Able to find solutions for administrative and logistical challenges related to schedules and distance	
**Unequivocal (U) and credible (C) findings:** Sustainability considerations (Martin et al., 2022) (U) Mentors recommended a continuation of interprofessional education in clinical placements (Naumann et al., 2020) (C) Introducing the Interprofessional Lens Early (Skinner, Simson, et al., 2021) (U) The learning activity must be simplified if more students are to be able to participate (Toth‐Pal et al., 2020) (U)	**Further development of interprofessional clinical practice** Able to be involved in developing and streamlining interprofessional clinical practice	
**Unequivocal (U) and credible (C) findings:** To use time to support the team to find structure in their collaboration (Conte et al., 2016) (U) The promotion of interprofessional collaboration between the students (Ericson et al., 2017) (U) The experience of providing students opportunities to work closely together by undertaking shared ‘team duties’ (Freeth et al., 2001) (U) To integrate students as members of the care team (Lait et al., 2011) (U) Supporting the teamwork (Carlsson et al., 2011) (U)	**Supporting students to work as a team** Able to support students to work as a team and integrate them as care team members	Competencies related to supporting the learning process in interprofessional clinical practice
***Not‐supported finding (NS), not included in meta‐aggregation:*** *Mentors need to serve as role models for students to learn them to work together as a team* (Ericson et al., 2017) *(NS)*		
**Unequivocal (U) and credible (C) findings:** Supporting students learning together (Dyar et al., 2019) (U) Getting the students to teach each other (Jacobsen et al., 2009) (U) To balance the learners' contributions in their shared reasoning (Conte et al., 2016) (U)	**Supporting shared learning among students** Able to support students to learn together and get them to teach each other	
***Not‐supported findings (NS), not included in meta‐aggregation:*** *To maintain common motivation* (Conte et al., 2016) *(NS)*		
**Unequivocal (U) and credible (C) findings:** To encourage the teams to make joint decisions based on common experiences or, if there were gaps in their understanding, to seek help from others (Conte et al., 2016) (U) Supporting students' initiative (Grace et al., 2016) (U) A challenging task to stand back to enable the students to learn (Jakobsen, 2016) (U) Encouraging students to be autonomous in their problem solving and offering them little direction, assuming the students would learn from experiencing the consequences of their decisions (Reeves et al., 2002) (U) Facilitating trust and independence (Jensen et al., 2023) (U) Offering students more input (Reeves et al., 2002) (C) The strategies to expand the teams' reasoning by balancing listening with encouragement and asking open questions (Conte et al., 2016) (U) Supporting student‐led learning (Dyar et al., 2019) (U) Student activating effectively (Toth‐Pal et al., 2020) (U) ***Not‐supported findings (NS), not included in meta‐aggregation:*** *A balance of learning directly from the clinical educator while being able to ask questions to confirm students' approach* (Chipchase et al., 2012) *(NS)* *To provide a learning environment where the students could work more autonomously* (Reeves & Freeth, 2002) *(NS)* *The students are given the responsibility of running the ward (of course, after sufficient tutoring) (*Jacobsen et al., 2009 *) (NS)* *As a part of the teaching, there is an expectation from the clinical tutors that the students are in front and take professional responsibility* (Jacobsen et al., 2009) *(NS)*	**Supporting the self‐efficacy of students and student teams** Able to stand back and encourage student teams' autonomy, initiative and to make joint decisions	
**Unequivocal (U) and credible (C) findings:** The students developed both in their professional and their interprofessional roles (Toth‐Pal et al., 2020) (U) Each student needs a supervisor of their profession present all the time (Chipchase et al., 2012) (U) Facilitating professional understanding (Carlsson et al., 2011) (U) Setting up for success – balancing challenges with opportunities; mentors raised concerns when mentoring students from another profession about how to determine the students' level of clinical knowledge and the appropriateness of any contributions the students may make the client care (Skinner, Simson, et al., 2021) (U) Introducing the interprofessional lens early was seen as important by participants, as it set the scene for future clinical experiences, and enabled students to have a better understanding of how the health system works and the differing roles of each health professional (Skinner, Simson, et al., 2021) (U)	**Supporting students' professional growth** Able to support student's professional and interprofessional skills and expertise	
***Not‐supported finding (NS), not included in meta‐aggregation:*** *To emphasize seeing tasks from one's own professional viewpoint, the students have to think more widely about their profession and tasks than they normally would* (Jacobsen et al., 2009) *(NS)*		
**Unequivocal (U) and credible (C) findings:** To centre the student's learning around a ‘multidisciplinary patient’ (Lait et al., 2011) (U) Finding the right patient was a key factor, but it turned out to be difficult (Toth‐Pal et al., 2020) (U) Setting patients' needs on the agenda for what the students are going to work with during the day (Jacobsen et al., 2009) (U) ***Not‐supported finding (NS), not included in meta‐aggregation:*** *Types of patients and possibilities for learning* (Jakobsen & Hansen, 2014) *(NS)*	**Supporting people‐centeredness in interprofessional mentoring** Able to mentor in a people‐centred way, identify and choose eligible patients for interprofessional clinical practice	
**Unequivocal (U) and credible (C) findings:** Illuminating interprofessional learning opportunities (Jensen et al., 2023) (C) Finding opportunities to set up the students to discover interprofessional principles themselves (Grace et al., 2016) (U) To organize the activity required extensive planning, but there were tricks to facilitate it (Toth‐Pal et al., 2020) (U) To support the task‐based approach in which the students were alone with the patients because it could stimulate authentic learning (Jakobsen et al., 2017) (U)	**Making use of interprofessional learning activities** Able to recognize and use unique and effective interprofessional learning opportunities and activities	
***Not‐supported findings (NS), not included in meta‐aggregation:*** *Some mentoring activities consisted of observation and discussion* (Lait et al., 2011) *(NS)* *To create a secure learning environment with authentic tasks (*Jacobsen et al., 2009 *) (NS)*		
**Unequivocal (U) and credible (C) findings:** To keep the team motivated when there was an imbalance between the complexity of the work and the team's level of experience (Conte et al., 2016) (U) Breaking down barriers (Carlsson et al., 2011) (U) Something other than expected happened (Jakobsen & Hansen, 2014) (U) Tension between uniprofessional and IPE components (Martin et al., 2022) (U)	**Managing challenges** Able to manage upcoming challenges; for example, breaking down hierarchical barriers and keeping the student team motivated when there is an imbalance between the complexity of the work and the team's level of experience or competence or unmotivated students. Mentors need to prepare for something other than expected to happen	
***Not‐supported finding (NS), not included in meta‐aggregation:*** *The experience of demanding concurrent responsibility for team mentoring and professional/individual mentoring* (Freeth et al., 2001) *(NS)*		
**Unequivocal (U) and credible (C) findings:** To set up regular interprofessional briefing sessions that focused on clinical and collaborative aspects (Chipchase et al., 2012) (U) The value of the debriefing sessions for interprofessional learning (Chipchase et al., 2012) (U) To create a safe environment and to guide the learners through the reflection (Conte et al., 2016) (U) When the reflections took place was important in relation to the specific situation being discussed (Conte et al., 2016) (U) Using a reflective approach (Carlsson et al., 2011) (U) To realize the necessity of reflecting on nursing (Jakobsen, 2016) (U) Helping the students to observe, analyse and reflect (Jakobsen, 2016) (U)	**Reflection and debriefing** Able to organize and hold interprofessional reflection and debriefing sessions, which focus on clinical and collaborative aspects.	
***Not‐supported finding (NS), not included in meta‐aggregation:*** *The experience of mentors considered the team reflection sessions to be of immense importance in providing excellent learning opportunities* (Freeth et al., 2001) *(NS)*		
**Unequivocal (U) and credible (C) findings:** Interprofessional assessment tool supported acknowledging the student journey (Skinner, Robson, & Vien, 2021) (U) Interprofessional assessment tool supported interprofessional authenticity (Skinner, Robson, & Vien, 2021) (U) Interprofessional assessment tool supported collective and collaborative learning (Skinner, Robson, & Vien, 2021) (U) A relevant working tool for assessment (Andermo et al., 2023) (U) A relevant assessment tool stimulated discussion about students' individual assessments (Andermo et al., 2023) (U) Setting up for success – balancing challenges with opportunities; supervising students from another profession would be particularly challenging in relation to student assessment if the focus was on discipline‐specific clinical reasoning (Skinner, Simson, et al., 2021) (U) Setting up for success – balancing challenges with opportunities; the participants suggested careful consideration be undertaken of the types of assessment that would be appropriate within an interprofessional context (Skinner, Simson, et al., 2021) (U)	**The use of interprofessional assessment tools** Able to use interprofessional assessment tools for student evaluation. Interprofessional assessment tools support students' collaborative learning and authenticity, acknowledge students' journey and stimulate students' individual assessment	
***Not‐supported finding (NS), not included in meta‐aggregation:*** *Difficulties in interpretation of specific concepts of a relevant assessment tool* (Andermo et al., 2023) *(NS)*		
**Unequivocal (U) and credible (C) findings:** The need for health organizations to allocate appropriate human and fiscal resources (Missen et al., 2012) (U) More time and privacy for students assessment (Andermo et al., 2023) (U) Alternating roles, presence and positioning of mentors (Jensen et al., 2023) (U) Problems of managing the demands of their ‘normal’ role and providing the students with sufficient levels of support in the training ward (Reeves et al., 2002) (U) A balance between patient care and role as mentor influenced mentors' motivation (Oosterbaad‐Lodder et al., 2023) (U) Creating student‐dedicated space (Dyar et al., 2019) (U) To provide continual facilitation for the student teams was ‘mentally straining’ (Reeves & Freeth, 2002) (U)	**The sustainable balance between different roles of interprofessional mentor** Able to allocate sufficient time and space for interprofessional mentoring and to balance the different roles of interprofessional mentor	Competencies related to creating an interprofessional mentor identity
***Not‐supported finding (NS), not included in meta‐aggregation:*** *Logistic challenges and support* (Jakobsen & Hansen, 2014) *(NS)*		
**Unequivocal (U) and credible (C) findings:** Interprofessional limitation (Dickie et al., 2019) (C) The experience of online resources which aimed to prepare students and mentors for interprofessional practice, supported identifying commonalities and respecting differences (Grace et al., 2016) (U) The experience of online resources which aimed to prepare students and mentors for interprofessional practice, supported learning from each other through cross‐disciplinary communication and interaction (Grace et al., 2016) (U) The project has opened for increased interprofessional collaboration in some cases (Toth‐Pal et al., 2020) (U) The learning ward broadened mentors' understanding of interprofessional collaboration (Reeves et al., 2002) (U) Ripple effects of the RIPES model (Martin et al., 2022) (U) Preceptor knowledge gaps related to curricula, goals and scope of practice of trainees from other professions, and communications style (Chen et al., 2016) (U)	**Interprofessional collaboration competence** Able to recognize and understand the meaning of interprofessional collaboration. To know the main tasks and competencies of the different professional groups. Able to respect different professions. Able to work in interprofessional collaboration	
***Not‐supported findings (NS), not included in meta‐aggregation:*** *Experience of interprofessional collaboration in the present clinical work varied* (Toth‐Pal et al., 2020) *(NS)* *The tutors are role models for the students, because the students watch the way the tutors cooperate as an interprofessional team (*Jacobsen et al., 2009 *) (NS)* *Although the students were encouraged to work closely in their teams, the facilitators from each profession tended to work in parallel, with little interprofessional collaboration* (Reeves & Freeth, 2002) *(NS)*		
**Unequivocal (U) and credible (C) findings:** To change supervision style in the interprofessional context (Chipchase et al., 2012) (U) Interprofessional clinical supervisor (IPCS) model development (Dickie et al., 2019) (C) Building a team of mentors (Ericson et al., 2017) (U) A variety of teaching approaches and levels of engagement with trainees of different professions (Chen et al., 2016) (U) Mentors' approach to learning (Dyar et al., 2019) (U) To provide interprofessional pre‐consultation instruction with both of the students and both of the mentors present (Jakobsen et al., 2017) (U)	**Commitment to the team of interprofessional mentors** Able to build a mentoring team with shared goals, a common teaching approach and an understanding of interprofessional clinical practice. Able to adopt the role of interprofessional mentor. Each mentor is committed to mentoring students from different professions and supporting each other's. *Able to give and get feedback on interprofessional mentoring from students, patients, and peers*	
***Not‐supported findings (NS), not included in meta‐aggregation:*** *The experience that each mentor has a particular mentoring style with their student teams* (Freeth et al., 2001) *(NS)* *The importance of getting to know each other* (Jakobsen & Hansen, 2014) *(NS)* *Participate on equal footing* (Jakobsen & Hansen, 2014) *(NS)* *To produce greater consistency in student team facilitation* (Reeves & Freeth, 2002) *(NS)* *The experience that mentors do not receive advice or feedback about their mentoring* (Freeth et al., 2001) *(NS)* *Learning together* (Dyar et al., 2019) *(NS)* *The lack of continuity in the mentoring team due to scheduling difficulties proved to be a major concern for all mentors* (Ericson et al., 2017) *(NS)* *To be aware of the importance of being open‐minded and reflective* (Jacobsen et al., 2009) *(NS)*		
**Unequivocal (U) and credible (C) findings:** Mentors value interprofessional education (Naumann et al., 2020) (U) Mentors found the allocated time for teaching stimulating to mentor committed students who were eager to learn and work in teams (Ericson et al., 2017) (U) Mentors got surprised because the students saw things that the mentors did not know about (Jakobsen, 2016) (U) Value of interprofessional education for students and patients (Naumann et al., 2020) (U) Perceived effectiveness of guidance influenced mentors' motivation (Oosterbaad‐Lodder et al., 2023) (U) Value of the RIPES placement model (Martin et al., 2022) (U) Feeling appreciated for your role influenced mentors' motivation (Oosterbaad‐Lodder et al., 2023) (U) The financial compensation for supervision was less than the reimbursement for care visits (Toth‐Pal et al., 2020) (U) Learning from others influenced mentors' motivation (Oosterbaad‐Lodder et al., 2023) (U) Tapping into unique possibilities included increased supervision capacity, learning more about other professions and a pathway into clinical supervision for junior clinicians in addition shared interprofessional placements may also open opportunities to expand placement capacity (Skinner, Simson, et al., 2021) (U) The supervisors experienced the activity as instructive and beneficial for patient care (Toth‐Pal et al., 2020) (U) Interprofessional assessments led to quality improvement for the patient (Toth‐Pal et al., 2020) (U)	**Motivation and attitudes towards interprofessional mentoring** Able to recognize the value of interprofessional education for students and patients. Interprofessional mentoring has increased the motivation to be a mentor. Able to feel appreciation for their role as an interprofessional mentor	
***Not‐supported findings (NS), not included in meta‐aggregation:*** *To be positive about the prospect of working on the interprofessional ward* (Reeves & Freeth, 2002) *(NS)* *Working on the training ward would contribute to mentors' personal, professional and academic development* (Reeves & Freeth, 2002) *(NS)*		
